# Potential of Snapshot-Type Hyperspectral Imagery Using Support Vector Classifier for the Classification of Tomatoes Maturity

**DOI:** 10.3390/s22124378

**Published:** 2022-06-09

**Authors:** Byeong-Hyo Cho, Yong-Hyun Kim, Ki-Beom Lee, Young-Ki Hong, Kyoung-Chul Kim

**Affiliations:** Department of Agricultural Engineering, National Institute of Agricultural Sciences, Jeonju 54875, Korea; cho2519@korea.kr (B.-H.C.); kyh39612@korea.kr (Y.-H.K.); keywii@korea.kr (K.-B.L.); sanm70@korea.kr (Y.-K.H.)

**Keywords:** support vector classifier (SVC), hyperspectral imagery, tomato maturity, PCA

## Abstract

It is necessary to convert to automation in a tomato hydroponic greenhouse because of the aging of farmers, the reduction in agricultural workers as a proportion of the population, COVID-19, and so on. In particular, agricultural robots are attractive as one of the ways for automation conversion in a hydroponic greenhouse. However, to develop agricultural robots, crop monitoring techniques will be necessary. In this study, therefore, we aimed to develop a maturity classification model for tomatoes using both support vector classifier (SVC) and snapshot-type hyperspectral imaging (VIS: 460–600 nm (16 bands) and Red-NIR: 600–860 nm (15 bands)). The spectral data, a total of 258 tomatoes harvested in January and February 2022, was obtained from the tomatoes’ surfaces. Spectral data that has a relationship with the maturity stages of tomatoes was selected by correlation analysis. In addition, the four different spectral data were prepared, such as VIS data (16 bands), Red-NIR data (15 bands), combination data of VIS and Red-NIR (31 bands), and selected spectral data (6 bands). These data were trained by SVC, respectively, and we evaluated the performance of trained classification models. As a result, the SVC based on VIS data achieved a classification accuracy of 79% and an F1-score of 88% to classify the tomato maturity into six stages (Green, Breaker, Turning, Pink, Light-red, and Red). In addition, the developed model was tested in a hydroponic greenhouse and was able to classify the maturity stages with a classification accuracy of 75% and an F1-score of 86%.

## 1. Introduction

Tomato (*Solanum lycopersicum*) is one of the most popular fruits in the world and is commercially valuable. It produced approximately 186.8 million tons worldwide in 2020 and is predominantly produced in China, India, USA, and Turkey [1]. Tomatoes, as a climacteric fruit, continue to both ripen after harvest and experience physiological changes [2]. For this reason, it is important to know the maturity stage of tomatoes, because harvesting can be changed depending on the different purposes such as priority of transportation to market and storage [3].

However, tomato farms are having difficulty growing and harvesting tomatoes, because of the aging of farmers, the reduction in agricultural workers as a proportion of the population, COVID-19, and so on. For this reason, several studies have been focused on converting to automation in a hydroponic greenhouse. In particular, several studies on agricultural robots, such as monitoring, transport, harvesting robots, and so on, are attractive as one of the ways for automation conversion in a hydroponic greenhouse. However, for developing agricultural robots, crop monitoring techniques will be necessary. A crop monitoring technique is a very useful tool for real-time decision support systems in agriculture, and in recent years, several research studies in the field of agriculture have been trying several approaches for applying the crop monitoring technique. In particular, the image processing methods using digital images [2,4], hyperspectral images [5,6], multi-spectral images [7,8], and so on, are widely used to develop the crop monitoring system, and these methods have been known as powerful tools for monitoring of various agricultural products. In particular, several studies have been conducted to classify the tomato maturity based on digital images [9,10], spatially resolved spectra [11], and so on, but most studies were considered at the laboratory level.

Hyperspectral imagery based on the line scan method can provide a lot of information both from the spectral and spatial domain by combining the traditional imaging and the spectroscopy methods, and providing a three-dimensional image with one spectral dimension and two spatial dimensions [12]. Line scan-based hyperspectral imagery is commonly used to obtain 3-D hyperspectral images and has been intensively developed for food and agricultural applications for the past 20 years [13,14]. Zhang et al. [15] used hyperspectral imagery data to predict the sugar content in pear and reported that was able to predict the sugar content of pears with an R value (correlation coefficient) of 0.897. Li et al. [16] investigated soluble solids content, and pH of cherry fruit by NIR hyperspectral imagery, and reported that the developed model achieved an accuracy of 96.4% for predicting the quality of cherry. However, it is difficult to handle the line scan-based hyperspectral imagery because there are more than 100 spectral bands. In addition, it is slow to acquire images in the line-scanning approach, and the line scan-based hyperspectral camera is large and expensive. Meanwhile, the snapshot hyperspectral sensor is a relatively new hyperspectral image sensor, and it offers certain advantages, such as acquiring hyperspectral images at video rate, ultra-portability, and easy handling with a small number of spectral bands [17]. For these reasons, some studies have used snapshot-type hyperspectral imagery in the agricultural sector, such as vegetation [18], fruit and vegetable [19], and species in meat [17]. However, there are only a few studies to classify the fruit ripeness/maturity using snapshot-type hyperspectral imagery.

The support vector machine is a machine learning method that can be used for both classification and regression and has been used in a variety of applications. In particular, the support vector classifier (SVC) has been applied to various fields, such as the classification of strawberry ripeness [20], recognition and classification of plants [21], and fruit classification [22].

Therefore, we aimed to develop a classification model of tomato maturity using snapshot-type hyperspectral imagery and SVC. We obtained tomato images using a snapshot-type hyperspectral camera at a laboratory level, and then the spectral data was trained by SVC. In addition, the developed model was examined to be applied to crop monitoring systems in a hydroponic greenhouse through the field test.

## 2. Materials and Methods

### 2.1. Sampling

“Dafnis” variety tomatoes harvested from January to February 2022 were used as a sample in this study and were harvested from a hydroponic greenhouse in South Korea. A total of 240 tomatoes were harvested into six maturity stages according to the USDA (United States Department of Agriculture) standard classification [23]. The tomato maturity stages were classified based on a* value, and the a* value of tomato skin was measured with a portable colorimeter (CR-20, KONICA MINOLTA, Tokyo, Japan) [24]. The a* value expresses the red/green scale and ranges from −127 to 127 (positive means red, negative means green). Table 1 shows the maturity stages of the tomatoes.

### 2.2. Hyperspectral Image Acquisition System

**Figure 1:**
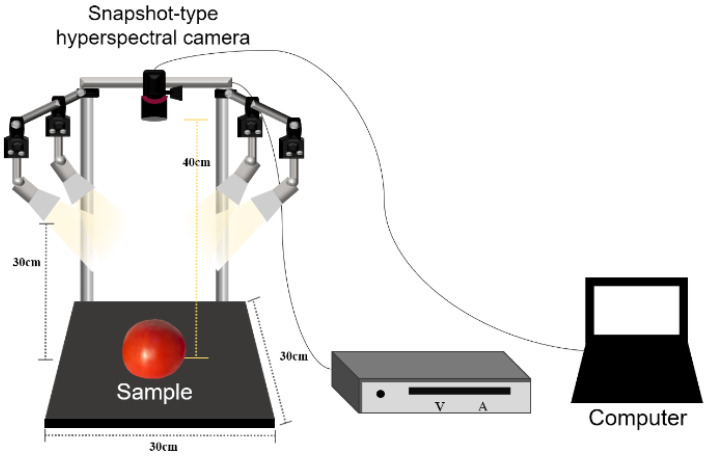
The schematic diagram of hyperspectral image acquisition system.

The schematic diagram of the hyperspectral image acquisition system is shown in Figure 1, and the system consisted of three components: (1) an imaging and lighting system, (2) a supporting frame, and (3) a computer. Tomato samples were uniformly illuminated using four halogen lamps (20 W, 12 V), and the halogen lamps were fixed at 300 mm above the bottom of the supporting frame. The snapshot-type hyperspectral cameras (SM4X4-VIS3, IMEC, Leuven, Belgium; SM4X4-RN2, IMEC, Leuven, Belgium) were used to take images of tomatoes and were fixed at a vertical distance of 400 mm above the bottom of the supporting frame. The hyperspectral cameras used in this study can acquire images in VIS and Red-NIR bands, respectively, and the specifications of each camera are summarized in Table 2. In addition, this system was controlled by the HIS Mosaic software (Ver. 5.0.2, IMEC, Leuven, Belgium).

### 2.3. Pre-Processing and Extraction

We acquired the white and dark reference images to correct the raw images from several effects, such as the noise generated by the device and uneven light source intensities. The white reference was acquired using a 95% white reference board (SG3151-U, IMEC, Leuven, Belgium), and the dark reference was acquired with the light source turned off and the camera lens completely covered with a lens cap.

ENVI software (Ver. 5.3, Exelis Visual Information Solutions Inc., Boulder, CO, USA) was used for hyperspectral image processing and spectra data extraction, and Otsu’s threshold method was applied to remove the background from the calibrated hyperspectral images. A photochemical reflectance index (PRI) and 625 nm band were applied to extract the spectra data of the tomato surface from obtained hyperspectral images using VIS and Red-NIR cameras, respectively [25]. The PRI was calculated using Formula (1), and $Ref_{588}$ and $Ref_{508}$ mean the reflectance of 588 nm and 508 nm bands, respectively.

(1) $$\begin{array}{l} PRI=\frac{Ref_{588}-Ref_{508}}{Ref_{588}+Ref_{508}} \end{array}$$

**Figure 2:**
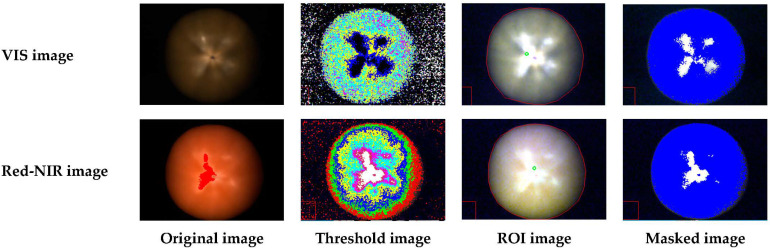
The pre-processing steps for hyperspectral image.

In addition, the region-of-interest (ROI) step was performed for removing any dead pixels, and the ROI was manually selected for each tomato image. Figure 2 shows the processing steps of the hyperspectral image.

## 3. Data Analysis

**Figure 3:**
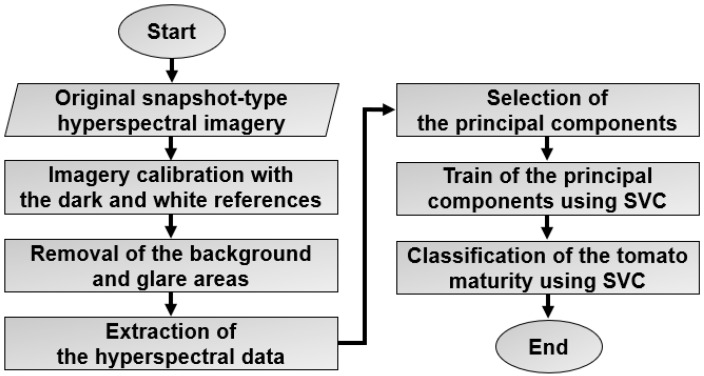
The flowchart diagram for classifying the tomatoes’ maturity stages from the snapshot-type hyperspectral imagery.

In this study, the principal component analysis (PCA) method was used to process the spectra data of tomatoes. The support vector classifier (SVC) model was used to analyze the processed spectra data by PCA, and the SVC model was implemented using the Scikit-learn machine learning library in a Python program [26]. Figure 3 shows the flowchart for classifying the tomatoes’ maturity stages using the snapshot-type hyperspectral imagery and SVC.

### 3.1. Principal Component Analysis (PCA)

PCA is a useful data reduction technique and is known as a pre-processing technique in hyperspectral imaging for different purposes [27]. Most of the studies that used PCA to analyze hyperspectral imaging, focused on ways of obtaining effective image classification or prediction [28,29]. In addition, PCA detects early process changes that might not be apparent from analyzing pieces of data individually. In this study, we investigated the principal component with high-retention explained variance to classify the maturity stages of tomatoes.

### 3.2. Support Vector Classifier (SVC)

The SVC methodology was conceived for binary classification of objects based on the training data, and multi-class SVC is commonly implemented by combining several binary SVC [30,31]. The SVC has a faster classification along with better accuracy compared with the other machine learning algorithms. In addition, it is able to handle high dimensional data based on a nonlinear model [32]. For these reasons, there are many studies using the SVC for classification in the agricultural sectors [20,21,22]. Therefore, we considered that the SVC is suitable for real-time classification of tomatoes’ maturity. Thus, the multi-class SVC was considered to classify the maturity stages of tomatoes. In this study, a Gaussian radial basis function (rbf) was used as a kernel to investigate the nonlinear relationship between input and output data. In addition, the SVC has two hyperparameters such as C and gamma. The parameter C controls the magnitude of allowed training errors and determines the regularization strength. The parameter gamma controls the rbf kernel shape. The grid search method was used to find the optimal parameters of SVC to classify the maturity stages of tomatoes.

### 3.3. Model Evaluation

The entire dataset containing a total of 258 spectra data was randomly divided into the two datasets, the training set (80%, 206 spectra data) and the testing set (20%, 52 spectra data). In this study, leave-one-out cross-validation has been used to select the best model among SVC, and we considered modeling the extracted VIS (16 input data), Red-NIR (15 input data), and a combination of VIS and Red-NIR (31 input data). In addition, we considered that select the spectra data through the correlation analysis (6 input data). Thus, the performances of the models according to the four input data conditions were compared and evaluated. The developed models were verified by several actions, such as the classification accuracy and F1-score. These were calculated by Formulas (2) and (5), respectively.

(2) $$Accuracy\;\left(\%\right)\;=\;\frac{TP\;+\;TN}{TP\;+\;TN\;+\;FP\;+\;FN}\times 100$$

(3) $$Precision\;=\;\frac{TP}{TP\;+\;FP}$$

(4) $$Recall\;=\;\frac{TP}{TP\;+\;TN}$$

(5) $$F1-score\;=\;2\times \frac{Precision\;\times \;Recall}{Precision\;+\;Recall}$$

where, *TP* and *TN* are true positive, true negative, respectively. *FP* and *FN* are false positive, false negative, respectively.

### 3.4. Field Test

**Figure 4:**
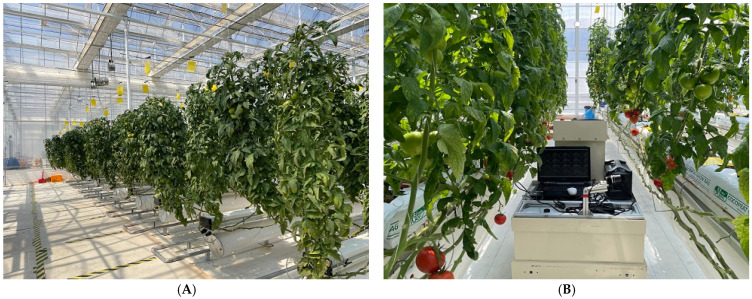
The actual image of the hydroponic greenhouse that we obtained tomato images (**A**) and the monitoring robot system used in this study (**B**).

Figure 4 shows the hydroponic greenhouse where we conducted the experiment and the monitoring robot system used in this study. We conducted the field test based on experimental results in the laboratory, and a hyperspectral camera that was selected through an indoor experiment was installed on the mobile robot. We acquired the dark reference image to correct the raw images from the noise generated by the device, and an 18% reference (LL LR1252, Manfrotto, NJ, USA) was used to correct the uneven light source intensities.

### 3.5. Statistics Analysis

Correlation analysis was conducted using bivariate analysis, and the Python program was used to analyze the significance in this study. The correlation between spectra data and the maturity stage of tomatoes was estimated using the Pearson correlation coefficient.

## 4. Results and Discussion

### 4.1. Spectra Data Analysis

**Figure 5:**
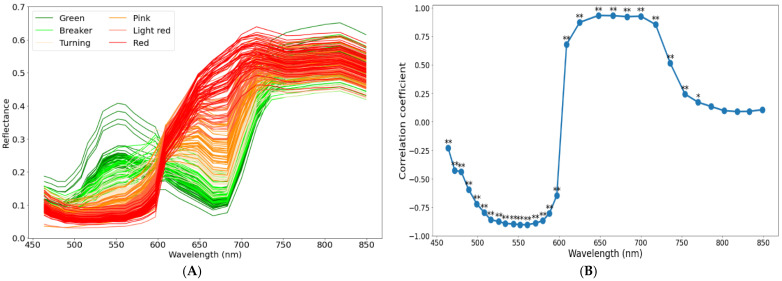
The spectral curves of six different maturity stages of the intact tomatoes (**A**) and the correlation coefficient between maturity stages and each waveband (**B**). ** and * Correlations are significant at the 0.01 and 0.05 levels, respectively.

The spectral curves in the spectral range of 460–850 nm containing 31 wavebands of the intact tomatoes at the different maturity stages were shown in Figure 5A, and this result showed a similar trend as that shown in previous studies [33,34]. As shown in Figure 5, the spectral differences of wavebands 516–580 nm and 625–718 nm were significant. The spectral reflectance of 516–580 nm decreased during the maturity process of tomatoes, while the spectral reflectance of 625–718 nm increased. These changes were probably caused by the progressive change in the color of the tomatoes from green to red. For example, the spectral reflectance of a near 670 nm is related to chlorophyll absorption of tomatoes. As a tomato ripens from green to red, its chlorophyll content would be reduced or disappear completely, and thus, the spectra reflectance at 666 nm and 683 nm also increased steadily [35]. In addition, the spectra reflectance of 400–550 nm is related to carotenoids, thus the spectra reflectance at 552 nm and 561 nm decreased steadily [36].

In addition, significant correlations were observed between maturity stages and most wavebands at the 99% level (*p* < 0.01) except for near 800 nm wavebands. We considered selecting several wavebands with a high correlation coefficient over 0.9 to develop a classification model for tomato maturity. As a result, a total of six wavebands, such as 552, 561, 648, 666, 683, and 700 nm, were selected.

**Figure 6:**
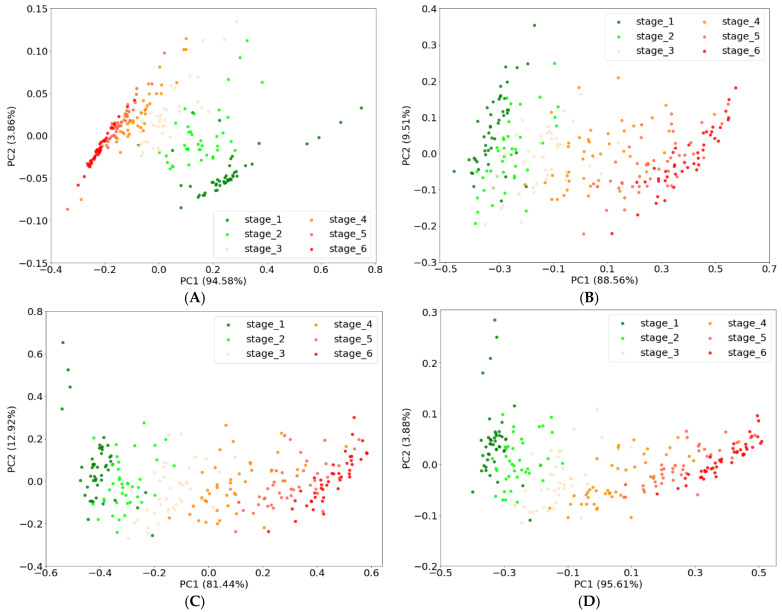
The PC1 and PC2 scores based on VIS data (**A**), Red-NIR data (**B**), combination data of VIS and Red-NIR data (**C**), and selected data from VIS and Red-NIR data (**D**).

PCA was conducted to visualize the differences between maturity stages from the spectra data of tomatoes. The five PCs (principal components) were determined, and the results of PCA according to input data were shown in Table 3. The explained variance of PC1 and PC2 reached over 94% in all conditions regardless of the input data. However, the rates of the contribution of PC3, PC4, and PC5 accounted for a very small part of the total. It means that PC1 and PC2 can explain the maturity stages of tomatoes. For this reason, the PC1 and PC2 scores of each maturity stage were plotted to observe the major distinction between them as shown in Figure 6. Although the score distribution map of tomatoes from the different maturity stages overlapped one another, tomatoes of the same maturity stage were more concentrated, forming different regions, regardless of the input data. However, the accurate classification of maturity stages was not easily achieved.

### 4.2. Classification Model

**Figure 7:**
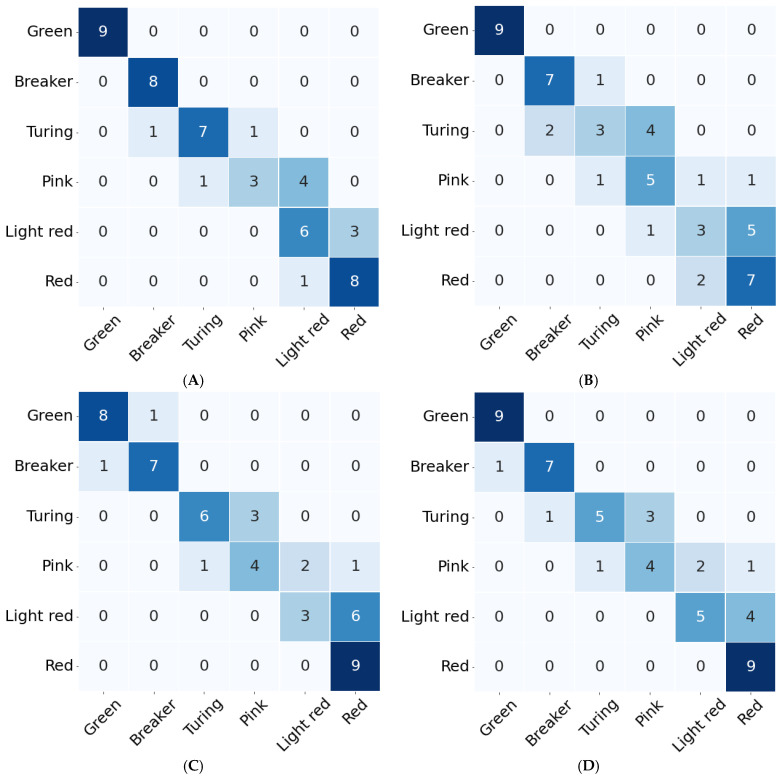
The confusion matrices of test set with VIS data (**A**), Red-NIR data (**B**), combination data of VIS and Red-NIR (**C**), and selected data from VIS and Red-NIR data (**D**).

Figure 7 shows the confusion matrices of the testing set with four different input data of SVC, and the optimal parameters and accuracy for each model are shown in Table 4. As shown in Figure 7 and Table 4, the accuracy of the VIS model was the highest at 79%, and it is considered that the accuracy of the VIS model including the red and green bands is the highest because of the skin color of tomatoes changes from green to red. In classifying the tomatoes’ maturity stage using the VIS model, the most accurate is on the Green stage which could be explained through the color percentage of green shades present on the tomatoes’ surface [37]. However, the model classified some fruits of the Pink stage as the Light-red stage, and it is considered to be because the Pink and Light-red stages were not well separated by the mix and the color gradient between them as shown in the PCA result (Figure 6A). These results showed a different trend to previous studies, and the other studies reported that the classification accuracy for Pink and Light-red stages was higher than that of Breaker and Turning stages [35,38]. It is considered to be due to various factors, such as harvest time of tomatoes, light source, device, and so on. In addition, all SVC models classified the Light-red stage as the Red stage, and this error occurred most frequently. This misclassification was caused by the colors of the Light-red visually appearing similar to Red as shown in Figure 6A. Meanwhile, all models except the Red-NIR model achieved more than 83% F1-score, and in particular, the VIS model achieved the best performance of 93% recall, 84% precision, and 88% F1-score. In this study, therefore, we identified the potential of a snapshot-type hyperspectral camera with VIS band as a non-destructive monitoring device for the maturity classification of tomatoes into the six stages.

### 4.3. Field Test

**Figure 8:**
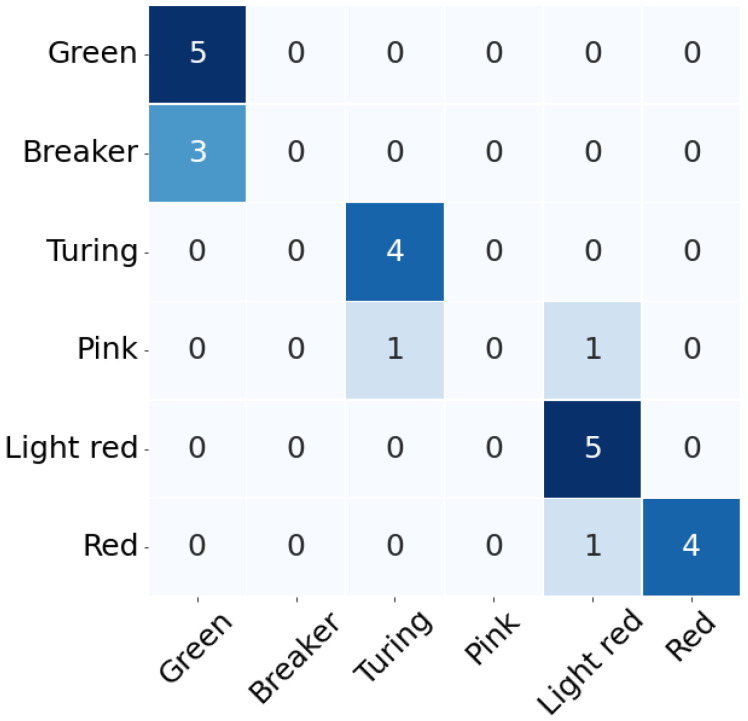
The confusion matrices of field test with VIS data.

As the above results, we considered conducting the field test using VIS hyperspectral camera. The VIS images of tomatoes containing a total of 24 samples were obtained in the hydroponic greenhouse (Figure 4) and were used to test the maturity classification model at the field level. Figure 8 shows the confusion matrix of the field test using the developed maturity classification model at the laboratory level. As shown in Figure 8, the classification accuracy was 75%, and recall, precision and F1-score were 78%, 95%, and 86%, respectively. Thus, we considered that this system can be used as a monitoring system in a tomato hydroponic greenhouse if the developed classification model based on snapshot-type hyperspectral imagery is applied to a mobile robot. It means that the system has the potential to be applied to other hydroponic fruits. However, the Breaker stage was classified as Green and the Pink stage was classified as Turing and Light-red stages. These results are considered to be due to several factors. First, tomatoes used for laboratory and field tests were grown at different times. Second, it was influenced by several test environments, such as film type of greenhouse, camera angle, and so on. The polyolefin film was used as the greenhouse film, and it is not scattering poly film. In addition, we acquired the hyperspectral images at the time when the external light was strongest. Thus, it will be necessary to carry out more experiments under various light conditions and seasons of cultivation to achieve more comprehensive classifications in the future.

## 5. Conclusions

We developed a classification model for tomato maturity into the six stages (Green, Breaker, Turning, Pink, Light-red, and Red) using SVC (Support vector classifier) model. Snapshot-type hyperspectral images of tomatoes containing VIS (460–600 nm) and Red-NIR (600–860 nm) bands were obtained at the laboratory level, and we selected several bands with a high relationship to tomato maturity. In addition, SVC models were trained using four different input data, such as VIS data (16 bands), Red-NIR data (15 bands), a combination of VIS and Red-NIR data (31 bands), and selected data (6 bands), and we evaluated the performance of maturity classification using several actions, such as classification accuracy, recall, precision and F1-score. The SVC based on VIS data achieved the classification accuracy of 79% and F1-score of 88%. As the above result, we conducted the field test in a hydroponic greenhouse, and the model could classify the maturity stages of tomatoes with a classification accuracy of 75% and an F1-score of 86%. Therefore, snapshot-type hyperspectral imagery aided by SVC (Support vector classifier) would be a useful tool to classify tomato maturity into six stages at the laboratory and field levels. In addition, this study demonstrated that a snapshot-type hyperspectral camera has the potential to be used as a tool to monitor the generative growth of tomatoes in a hydroponic greenhouse. Nevertheless, it will be necessary to carry out more experiments under various field conditions to achieve a more comprehensive classification in the future.

## Figures and Tables

**Table 1:** Maturity stages of tomato fruits.

Maturity	Description	a* Value
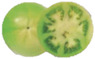	**Green;** Entirely green	−4.87 ± 2.73
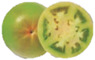	**Breaker;** First appearance of external pink or red color; not more than 10%	−0.33 ± 2.50
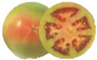	**Turning;** Over 10% but not more than 30% red or pink	4.98 ± 2.70
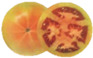	**Pink;** Over 30% but not more than 60% pinkish or red	10.70 ± 2.53
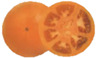	**Light red;** Over 60% but not more than 90% red	16.57 ± 2.32
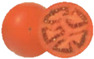	**Red;** Over 90% red	19.31 ± 1.60

**Table 2:** The hyperspectral camera specification.

Variable	Specification	
	VIS	Red-NIR
Sensor	AMS/CMOSIS CMV2000 mono	
Resolution	2048 × 1088, 2.2 MPixel	
Pixel size	5.5 μm	
Sensor size/diagonal	11.3 × 6.0 mm	
Optical size	2/3 “	
FPS	170 (USB3.0)	
Focal length	25 mm	
Exposure time	2.0 ms	1.3 ms
Wavelength range	460–600 nm	600–860 nm
Band: peak central wavelengths [nm]	464, 472, 480, 489, 499, 508, 516, 526, 534, 544, 552, 561, 571, 580, 588, 597	609, 625, 648, 666, 683, 700, 718, 736, 754, 770, 786, 802, 818, 833, 849

**Table 3:** The principal component analysis (PCA) results according to input data.

Data	% of Variance					
	PC1	PC2	PC3	PC4	PC5	Total
VIS	94.58	3.86	1.47	0.07	0.02	100
RN	88.56	9.51	1.77	0.12	0.03	99.99
VN	81.44	12.92	3.27	1.90	0.28	99.81
SD	95.61	3.88	0.44	0.07	0.00	100

VIS: VIS data, RN: Red-NIR data, VN: combination of VIS and Red-NIR data, SD: selected data from VIS and Red-NIR data.

**Table 4:** The results of grid search and performance evaluation for SVC with input data.

Input Data	Hyperparameters		Performance for SVC			
	C	Gamma	Accuracy	Recall	Precision	F1-Score
VIS	150	5	79%	93%	84%	88%
RN	150	5	65%	85%	74%	79%
VN	150	5	71%	95%	74%	83%
SD	150	5	75%	93%	80%	86%

VIS: VIS data, RN: Red-NIR data, VN: combination of VIS and Red-NIR data, SD: selected data from VIS and Red-NIR data.

## Data Availability

The data presented in this study are available on request from the corresponding author. The data are not publicly available due to privacy reasons.
